# CHARACTERIZING END-OF-LIFE COMMUNICATION IN FAMILIES

**DOI:** 10.1093/geroni/igad104.2974

**Published:** 2023-12-21

**Authors:** Melanie Horning

**Affiliations:** Towson University, Towson, Maryland, United States

## Abstract

The chronic disease course can be uncertain, contributing to delayed end-of-life discussion within families resulting in missed opportunity to articulate wishes, increased decisional uncertainty, and delayed hospice care. Consistent with the Family Communication Patterns Theory (FCPT), family communication patterns may increase understanding of end-of-life discussion, hospice utilization, and may affect the experience of “a good death.” This cross-sectional study used a modified Revised Family Communication Pattern instrument (RFCP) to examine the family communication characteristics of 56 family members of loved ones who died from chronic illnesses while in hospice. Most families (42.9%) were pluralistic, reporting communication styles with high conversation and low conformity orientation; (39.29%) were protective, reporting low conversation and high conformity orientation. Pluralistic families had more end-of-life conversations than did protective families. This study’s findings support the idea that using the RFCP to assess familial communication patterns may be useful for end-of-life conversation and decision-making

